# Investigating Prescription Medication Expenditures and Level of Perceived Health Status among Older Adults with Pain in the United States

**DOI:** 10.3390/medicines9030017

**Published:** 2022-02-22

**Authors:** David R. Axon, Leonard P. Barrios

**Affiliations:** 1 Department of Pharmacy Practice and Science, College of Pharmacy, University of Arizona, Tucson, AZ 85721, USA; lobarrios@pharmacy.arizona.edu; 2 Center for Health Outcomes and Pharmacoeconomic Research, College of Pharmacy, University of Arizona, Tucson, AZ 85721, USA

**Keywords:** pain, self-perceived health, prescription medications, healthcare expenditures, older adults

## Abstract

**Background:** the objective of this retrospective, cross sectional study was to assess the relationship between different levels of perceived health status and prescription medication expenditures among United States adults aged ≥50 years with self-reported pain. **Methods:** using 2019 Medical Expenditure Panel Survey (MEPS) data, four levels of perceived health status were formed (excellent, very good, good, and fair/poor health). Differences between characteristics in the groups were compared using chi square tests. Adjusted linear regression models, using logged positive annual prescription medication expenditures, were constructed to assess differences in prescription medication expenditures between levels of perceived health. **Results:** a total of 4826 individuals were included in the study. Adjusted linear regression analysis indicated those who reported excellent health had 65.8% lower annual prescription medication expenditures than those with fair/poor health. Those with very good health had 49.7% lower annual prescription medication expenditures than those with fair/poor health, while those with good health had 27.2% lower annual prescription medication expenditures than those with fair/poor health. **Conclusions:** better perceived health status was generally associated with relatively lower prescription medication expenditures. Further research is needed to investigate if this pattern is also observed for other categories of healthcare expenditures and in other populations.

## 1. Introduction

Pain is a complex, subjective condition that is difficult to measure due to variation among individuals [1]. The International Association for the Study of Pain has defined pain as “an unpleasant sensory and emotional experience associated with, or resembling that associated with, actual or potential tissue damage,” [2]. Pain is a common condition that has become increasingly more prevalent in recent years and is associated with older age [3,4]. Pain is associated with many common diseases in later life, including dermatology, gastrointestinal, cardiovascular, pulmonary, rheumatology, endocrine, nephrology, immune, neurology, oncology, and miscellaneous complaints [5]. Pain is also one of the most common reasons for individuals to seek medical care [6,7]. For instance, individuals aged ≥65 years accounted for 30.8% of United States (US) adults with chronic pain in 2019 [3]. Another report from 2011 stated half of older adults had bothersome pain in the past month among 7601 US adults aged ≥65 years [8]. Another report found that over 100 million US adults had chronic pain in 2010, and the national cost of pain ranged from USD 560 billion to USD 635 billion (US dollars in 2010) [9,10]. 

Not only is pain management burdensome from an economic perspective, but individuals with pain often use an onerous quantity of different medications to help manage pain. For example, one study found that individuals with chronic pain used 13 different management strategies on average, many of which were prescription medications [11]. These prescription medications included the following drug classes: opioids, analgesics, anticonvulsants, antidepressants, antipsychotics, barbiturates, beta blockers, calcium channel blockers, muscle relaxants, non-steroidal anti-inflammatory drugs (NSAIDs), sedatives, steroids, triptans, and others [11]. Meanwhile, a recent literature review identified 16 different categories of prescription medications are used by community-dwelling individuals to help manage pain [12]. These categories included: prescription medications generally, analgesics, acetaminophen, anticonvulsants, antidepressants, anxiolytics/sedatives/antipsychotics, salicylates, beta/calcium channel blockers, disease-modifying anti-rheumatic drugs (DMARD)/steroids, any type of injections, muscle relaxants, NSAIDs, opioids, any type of topical product, triptans, and others [12]. Prescription medications are therefore one of the most common approaches for managing pain [11,12,13]. 

Total US spending on prescription medications amounted to USD 507.9 billion in 2019, which was a 5.4% increase from the prior year’s spending [14]. This number has been steadily rising over the last several years, for instance, there was a similar percent increase of 5.5% from 2017 to 2018 [15]. Reasons to explain this increase include new drugs, drug pricing changes, increasing numbers of patients, disease patterns, state and federal policies, and prescribing practices [15]. 

Although many characteristics are associated with pain in older adults, one characteristic of interest is self-reported perception of health [16]. Self-perceived health status is considered an important component to promote population health [17]. There is limited information that describes the association between perceived health status and prescription medication expenditures among older US adults with pain, yet it is possible that prescription medication expenditures may differ based on the perceived health status of older adults with pain. Therefore, the objective of this study was to assess the relationship between different levels of perceived health status and prescription medication expenditures among US adults aged ≥50 years with self-reported pain, hypothesizing that better perceived health status was associated with relatively lower prescription medication expenditures.

## 2. Methods

This was a retrospective, cross-sectional study using the 2019 Medical Expenditure Panel Survey (MEPS) data. The MEPS sampling framework is adapted from the National Health Interview Survey sampling framework, and weighting variables are provided by MEPS to produce nationally representative estimates of the non-institutionalized civilian US population during analysis [18]. MEPS has three main components, including the MEPS household component (MEPS-HC), MEPS insurance component (MEPS-IC), and MEPS medical provider component (MEPS-MPC) [18]. MEPS-HC data are collected by surveying eligible households five times over a two-year period, and include demographic and personal characteristics, health conditions, and health status, access to healthcare, healthcare service use, and healthcare expenditure data. Data from MEPS-IC and MEPS-MPC supplement MEPS-HC data to improve the validity and reliability of the data [18]. This study used the 2019 full-year consolidated data file (MEPS-HC-216), which contained data from MEPS panel 22 (interview rounds 3, 4, 5) and panel 3 (interview rounds 1, 2, 3) [19,20] and was the most up-to-date data available at the time of the study. All MEPS subjects provided verbal informed consent before data collection.

The study inclusion criteria were: MEPS subjects alive for the full 2019 calendar year; aged ≥50 years; reported pain that interfered with normal work (including both work outside the home and housework) in the past four weeks; and positive annual prescription medication healthcare expenditures. 

The dependent variable was annual positive prescription medication expenditures. The independent variable was self-perceived health status, categorized as excellent, very good, good, and fair/poor. Table 1 outlines the potential confounders that served as control variables. These included age (50–64, ≥65 years), sex (male, female), ethnicity (Hispanic, non-Hispanic), race (white, other), marital status (married, other), education status (up to high school, high school, more than high school), employment status (employed, unemployed), health insurance coverage (private, public, uninsured), poverty status (based on income: poor/near poor/low, middle/high), help with activities of daily living (yes, no), help with instrumental activities of daily living (yes, no), frequent exercise (yes, no), current smoker (yes, no), number of chronic conditions (≥5, <5), pain intensity (quite a bit/extreme, little/moderate), mental health status (excellent, very good, good, fair/poor), and US census region (Northeast, Midwest, South, West) [19,20]. 

Chi-square tests were used to identify statistical differences between groups. Because of the non-linear nature of expenditure data, logarithmically transformed data were used in the adjusted linear regression model to assess differences in prescription medication expenditures between health status categories, with fair/poor acting as the reference group. The adjusted model included the independent variable (perceived health status) and all the control variables. Analyses accounted for the complex MEPS design. Nationally representative estimates were obtained using the relevant weighting variable, and variance estimates were calculated using the Taylor-series linearization method. An alpha level of 0.05 was chosen a priori, and all analyses were conducted using the SAS Studio statistical software (SAS Institute Inc., Cary, NC, USA).

## 3. Results

There were 28,512 available subjects in the 2019 MEPS set of data, with 4826 meeting the eligibility criteria to be included in the study. The prevalence of excellent perceived health status was 8.1% 95% confidence interval (CI) 7.1, 9.0). Very good health status had a prevalence of 28.9% (95% CI 27.2, 30.6), good health status was 36.3% (95% CI 34.6, 38.0), and fair/poor health status was 26.8% (95% CI 25.2, 28.3). 

As shown in Table 2, the majority of the subjects included were at least 65 years and older (53.2%), female (55.1%), non-Hispanic (90.8%), white (81.9%), married (57.4%), had an education greater than high school (54.7%), unemployed (60.7%), private health insurance coverage (57.4%), middle/high income (69.7%), did not need help with activities of daily living (94.8%) or instrumental activities of daily living (91.1%), did not frequently exercise (57.8%), not current smokers (84.8%), had less than five chronic conditions (76.4%), and had little/moderate pain (75.2%). Participants most commonly reported having very good mental health (31.3%) and lived in the southern census region (33.8%). There was a significant difference between all characteristics except for age (*p* = 0.2666) and sex (*p* = 0.3689).

**Figure 1:**
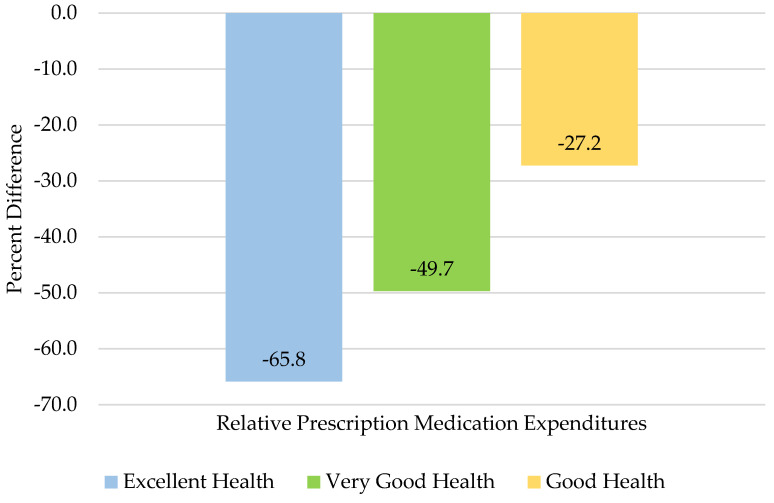
Percent difference of 2019 adjusted annual positive prescription medication expenditures for United States older adults (age ≥ 50 years) with pain in the past four weeks relative to individuals who reported having fair/poor health for those with excellent health (blue bar), very good health (green bar), and good health (yellow bar). The negative bars indicate those with excellent, very good, or good health have lower costs relative to those with fair/poor health.

The findings of the adjusted linear regression models are reported as a percent difference relative to those individuals who reported having fair/poor health. As shown in Figure 1, older US adults with pain who reported excellent health had 65.8% lower annual prescription medication expenditures than those who reported having fair/poor health. Those who reported having very good health had 49.7% lower annual prescription medication expenditures than those with fair/poor health, while those with good health had 27.2% lower annual prescription medication expenditures than those with fair/poor health.

## 4. Discussion

This study examined the association between self-perceived health status in older US adults with pain and prescription medication expenditures. The primary finding from this study was that the better older US adults with pain perceived their health status, the lower their relative prescription medications costs were compared to those with poorer perceived health status. Although these findings are perhaps unsurprising, there are no contemporary studies that assess the relationship between perceived health status and prescription medication costs, thus this study adds new information to the literature. However, previous studies have explored similar topics and reported findings that correlate with those in the current study. For instance, a 2003 study found that prescription medication expenditure was higher in older adults with fair (USD 1023) or poor (USD 1302) health status, compared to those who reported excellent/very good health status (USD 509) [21]. 

Other variables that were controlled for in the analysis may also explain some of these results, such as self-reported pain severity and number of comorbid conditions. A previous study using MEPS data found that over half (54.5%) of US older adults who reported having extreme or quite a bit of pain had fair or poor perceived health, whereas only 17.8% of those with moderate or little pain had fair or poor perceived health [22]. Compared to those with little pain, older US adults (≥50 years of age) had 32% greater prescription medication costs if they reported having extreme pain, 35% greater costs if they reported quite a bit of pain, and 29% greater costs if they reported having moderate pain [23].

The presence of multiple comorbid conditions may also have an impact on prescription medications costs, considering that the current study found that of those with fair/poor health status, 40.8% had ≥5 chronic conditions. Several other factors in the MEPS dataset are known to be associated with having multiple (≥5) chronic conditions including age, gender, ethnicity, race, employment status, functional limitations, work limitations, pain severity, and perceived health status [24]. Another study that also used MEPS data demonstrated that prescription medication expenditures were over 100% greater among older US adults with pain and multimorbidity (≥2 chronic conditions), versus those with no multimorbidity (<2 chronic conditions) [25]. The same study also identified that prescription medication expenditures had the greatest costs compared to several other categories of costs examined (including hospital inpatient, office-based, home health care, and total healthcare costs) [25], which emphasizes the importance of considering prescription medication costs relative to other costs.

The findings of this study may have implications for the management of pain among older US adults. However, it is important to note that these findings should not discourage appropriate prescription medication use. Prescription medications are typically prescribed to improve the health of an individual, either to help prevent a condition from developing or worsening, or to treat an existing condition. Traditionally, pain has been managed through pharmacological treatment, i.e., analgesics [13]. For example, the World Health Organization’s analgesic ladder is a standard model in pain management therapy consisting of three steps, starting with non-opioid analgesics for mild pain, up to potent opioids for severe pain [26]. 

However, opioids are sometimes not a desirable option given that they may be inappropriately prescribed or withdrawn abruptly as healthcare providers try to address the opioid epidemic in the US [27]. There is also a cost associated with opioid use; a recent MEPS study reported that older US adults (≥50 years) with pain who used at least one opioid in 2015 had 63% greater prescription medication costs that those who did not use opioids [28].

There may be opportunities to optimize prescribing that could reduce prescription medication costs or consider non-pharmacological options, as appropriate. Literature reviews have identified several non-pharmacological therapies that exist for pain management. For example, one systematic review found that strategies such as multidisciplinary rehabilitation, massage, acupuncture, and yoga have been reported among adults with low back pain [29].

Another systematic review of 18 studies among community dwelling adults with pain reported the following non-pharmacological pain management strategies: consulted medical practitioner, chiropractor, surgery, activity modification or restriction, acupuncture, altering the body potion or posture, using an assistive device, exercise, hot/cold modalities, massage, physical therapy, transcutaneous electrical nerve stimulation (TENS), prayer or medication, relaxation, rest or sleep, therapy, complementary and alternative medicine, dietary and herbal supplements, diet modification, and others [12]. Of these, exercise and massage were among the most commonly used [12]. 

Studies have investigated the association between frequent exercise and healthcare expenditures. For example, a recent MEPS study found that US adults aged ≥50 years who did at least 30 min of moderate-vigorous intensity physical activity at least five times a week had 15% lower annual prescription medication expenditures in 2018 compared to those who did not meet this level of exercise [30]. 

With regards to massage, a recent randomized controlled trial of patients with low back pain received deep tissue massage or deep tissue massage with NSAIDs. The trial found that deep tissue massage had a positive effect on reducing pain among patients with chronic low back pain [31].

Non-pharmacological therapies have shown moderate effectiveness for chronic low back pain at the short and intermediate term, but most of their effects independently were small and with little long-term evidence [32]. Though additional investigation is needed, several other forms of non-pharmacological treatment may be more effective as a potential adjunct to prescription drugs [33]. Non-pharmacological treatment may not act as a replacement for prescription medications, but rather serve to complement medications to help manage pain and reduce reliance on medications [34,35]. 

Previous research as investigated the association of various interventions on health status. For instance, one study of older women in Spain who received motivational aquatic resistance training found that several factors, including greater satisfaction, self-selection, volition, and autonomy, were associated with greater well-being, adherence, and health outcomes [36]. Another study found traditional Chinese medicine, such as acupuncture, herbal medicine, and dietary therapy was perceived to improve health and reduce the stress and side effects of treatments among cancer survivors in Australia [37].

Advantages of this study included the large nationally representative sample of US adults with pain, and the availability of data for many potentially confounding variables that could be controlled for in analyses.

Limitations of the study included the self-reported nature of the MEPS data, which could lead to biases. Variables in the study were a combination of objective measures and subjective measures; although the subjective variables allowed for the inclusion of patient-reported data, they may also have led to biases. To be included in the study, individuals needed to have positive annual prescription medication expenditure, thus older individuals that had pain but did not have positive prescription medication expenditures were not captured in the results. The study also did not include institutionalized or non-civilian individuals; thus, the findings cannot be generalized to that population. 

Greater effort is therefore needed to help improve the perceived health status of older US adults with pain to help reduce their need for prescription medications, and further research is needed to assess strategies that may help improve pain management without adversely affecting health outcomes. 

## 5. Conclusions

The adjusted linear regression analyses in this retrospective, cross-sectional study found that relative to fair/poor perceived health status, excellent perceived health status was associated with lowest prescription medication expenditures, followed by very good and good perceived health status. The findings from this study add contemporary information to the literature about the ever-increasing prescription medication costs in the US. These findings also suggest that more effort is warranted to help improve the health of older US adults with pain to help reduce their need for prescription medications, and there is a need for additional research to assess strategies that may help improve pain management without adversely affecting health outcomes.

## Figures and Tables

**Table 1:** Potential confounding variables that served as control variables in the adjusted linear regression analysis.

Variable	Variable Levels
Age (years)	50–64
	≥65
Sex	Male
	Female
Ethnicity	Hispanic
	Non-Hispanic
Race	White
	Other
Marital status	Married
	Other
Education status	Up to high school
	High school
	More than high school
Employment status	Employed
	Unemployed
Health insurance coverage	Private
	Public
	Uninsured
Poverty status	Poor/near poor/low income
	Middle/high income
Help with ADL	Yes
	No
Help with IADL	Yes
	No
Frequent exercise	Yes
	No
Current smoker	Yes
	No
Number of chronic conditions	≥5
	<5
Pain intensity	Quite a bit/extreme
	Little/moderate
Mental health status	Excellent
	Very good
	Good
	Fair/poor
US census region	Northeast
	Midwest
	South
	West

Abbreviations: ADL = activities of daily living, IADL = instrumental activities of daily living, US = United States.

**Table 2:** Sample characteristics of older United States adults with self-reported pain in the past four weeks, stratified by self-reported perceived health status.

Characteristics	Excellent Health (*N* = 351) % (95% CI)	Very Good Health (*N* = 1295) % (95% CI)	Good Health (*N* = 1760) % (95% CI)	Fair/Poor Health (*N* = 1420) % (95% CI)	*p*
Age (years)					0.2666
50–64	46.2 (40.2, 52.2)	48.1 (44.7, 51.4)	44.6 (41.6, 47.6)	48.5 (45.1, 52.0)	
≥65	53.8 (47.8, 59.8)	51.9 (48.6, 55.3)	55.4 (52.4, 58.4)	51.5 (48.0, 54.9)	
Sex					0.3689
Male	48.4 (42.1, 54.7)	46.2 (42.9, 49.5)	43.7 (41.5, 46.0)	44.1 (41.3, 46.9)	
Female	51.6 (45.3, 57.9)	53.8 (50.5, 57.1)	56.3 (54.0, 58.5)	55.9 (53.1, 58.7)	
Ethnicity					<0.0001
Hispanic	6.2 (3.6, 8.8)	5.5 (4.0, 7.0)	10.4 (8.5, 12.3)	12.5 (9.9, 15.1)	
Non-Hispanic	93.8 (91.2, 96.4)	94.5 (93.0, 96.0)	89.6 (87.7, 91.5)	87.5 (84.9, 90.1)	
Race					0.0002
White	85.3 (80.8, 89.8)	85.7 (83.6, 87.8)	80.1 (77.7, 82.6)	79.3 (76.2, 82.4)	
Other	14.7 (10.2, 19.2)	14.3 (12.2, 16.4)	19.9 (17.4, 22.3)	20.7 (17.6, 23.8)	
Marital status					<0.0001
Married	69.0 (63.6, 74.4)	62.0 (58.7, 65.4)	58.1 (55.4, 60.8)	48.0 (44.6, 51.4)	
Other	31.0 (25.6, 36.4)	38.0 (34.6, 41.3)	41.9 (39.2, 44.6)	52.0 (48.6, 55.4)	
Education status					<0.0001
Up to high school	9.2 (5.5, 12.9)	5.8 (4.5, 7.2)	16.1 (14.0, 18.2)	23.4 (20.2, 26.5)	
High school	22.2 (17.3, 27.1)	28.5 (25.4, 31.6)	32.3 (29.8, 34.8)	33.7 (30.6, 36.7)	
More than high school	68.6 (62.7, 74.4)	65.6 (62.2, 69.0)	51.6 (48.7, 54.5)	42.9 (39.5, 46.4)	
Employment status					<0.0001
Employed	48.8 (42.7, 54.9)	50.0 (46.8, 53.1)	39.9 (37.0, 42.8)	24.3 (21.6, 27.0)	
Unemployed	51.2 (45.1, 57.3)	50.0 (46.9, 53.2)	60.1 (57.2, 63.0)	75.7 (73.0, 78.4)	
Health insurance coverage					<0.0001
Private	67.4 (61.2, 73.5)	70.2 (67.2, 73.1)	55.8 (52.8, 58.8)	42.8 (39.3, 46.2)	
Public	31.3, (25.3, 37.4)	28.2 (25.3, 31.2)	40.4 (37.5, 43.3)	54.4 (51.2, 57.7)	
Uninsured	1.3 (0.1, 2.5)	1.6 (0.9, 2.3)	3.8 (2.6, 5.0)	2.8 (1.7, 3.9)	
Poverty status					<0.0001
Poor/near poor/low income	18.3 (13.6, 23.0)	18.3 (15.9, 20.8)	32.2 (29.2, 35.2)	44.1 (40.5, 47.8)	
Middle/high income	81.7 (77.0, 86.4)	81.7 (79.2, 84.1)	67.8 (64.8, 70.8)	55.9 (52.2, 59.5)	
Help with ADL					<0.0001
Yes	1.6 (0, 4.1)	1.2 (0.5, 1.8)	3.8 (2.7, 4.9)	12.4 (10.2, 14.6)	
No	98.4 (95.9, 100.0)	98.8 (98.2, 99.5)	96.2 (95.1, 97.3)	87.6 (85.4, 89.8)	
Help with IADL					<0.0001
Yes	3.3 (0.5, 6.1)	2.5 (1.6, 3.4)	7.4 (5.9, 8.9)	19.5 (16.8, 22.1)	
No	96.7 (93.9, 99.5)	97.5 (96.6, 98.4)	92.6 (91.1, 94.1)	80.5 (77.9, 83.2)	
Frequent exercise					<0.0001
Yes	64.6 (58.8, 70.3)	53.9 (50.9, 56.9)	39.5 (36.8, 42.3)	26.4 (23.5, 29.3)	
No	35.4 (29.7, 41.2)	46.1 (43.1, 49.1)	60.5 (57.7, 63.2)	73.6 (70.7, 76.5)	
Current smoker					<0.0001
Yes	8.2 (4.9, 11.4)	8.7 (6.9, 10.5)	16.5 (14.5, 18.4)	22.4 (19.3, 25.6)	
No	91.8 (88.6, 95.1)	91.3 (89.5, 93.1)	83.5 (81.6, 85.5)	77.6 (74.4, 80.7)	
Number of chronic conditions					<0.0001
≥5	10.3 (5.8, 14.8)	11.9 (10.2, 13.7)	23.1 (20.8, 25.4)	40.8 (38.2, 43.4)	
<5	89.7 (85.2, 94.2)	88.1 (86.3, 89.8)	76.9 (74.6, 79.2)	59.2 (56.6, 61.8)	
Pain intensity					<0.0001
Quite a bit/extreme	7.6 (3.2, 12.1)	11.1 (9.2, 13.0)	21.0 (18.7, 23.2)	49.8 (46.6, 53.1)	
Little/moderate	92.4 (87.9, 96.8)	88.9 (87.0, 90.8)	79.0 (76.8, 81.3)	50.2 (46.9, 53.4)	
Mental health status					<0.0001
Excellent	70.2 (64.9, 75.5)	24.7 (21.7, 27.6)	13.9 (11.9, 16.0)	9.1 (7.1, 11.0)	
Very good	19.6 (15.0, 24.2)	57.0 (53.9, 60.1)	26.7 (24.5, 28.8)	13.5 (11.4, 15.6)	
Good	7.7 (3.7, 11.7)	14.9 (12.4, 17.4)	52.2 (49.6, 54.9)	37.1 (33.8, 40.3)	
Fair/poor	2.6 (1.1, 4.0)	3.4 (2.3, 4.6)	7.2 (5.7, 8.7)	40.3 (37.3, 43.4)	
US census region					<0.0077
Northeast	13.2 (8.4, 17.9)	15.8 (12.6, 19.1)	17.9 (14.5, 21.3)	18.2 (14.8, 21.7)	
Midwest	18.0 (12.8, 23.2)	25.1 (22.1, 28.2)	22.8 (19.4, 26.2)	21.6 (18.8, 24.4)	
South	37.9 (31.3, 44.6)	35.7 (31.4, 40.1)	36.2 (32.8, 39.6)	40.4 (36.6, 44.3)	
West	30.9 (24.7, 37.1)	23.3 (19.7, 26.8)	23.1 (19.5, 26.7)	19.7 (16.6, 22.9)	

Abbreviations: % = percentage, CI = confidence interval, ADL = activities of daily living, IADL = instrumental activities of daily living, US = United States. Statistical differences between groups identified using chi-square tests.

## Data Availability

The data presented in this study are available on request from the corresponding author.

## Abbreviations

ADL	activities of daily living
CI	confidence interval
DMARD	disease-modifying anti-rheumatic drugs
IADL	instrumental activities of daily living
MEPS	Medical Expenditure Panel Survey
MEPS-HC	Medical Expenditure Panel Survey household component
MEPS-IC	Medical Expenditure Panel Survey insurance component
MEPS-MPC	Medical Expenditure Panel Survey medical provider component
NSAIDs	non-steroidal anti-inflammatory drugs
TENS	transcutaneous electrical nerve stimulation
US	United States

## References

[1] Younger J., McCue R., Mackey S. (2009). Pain outcomes: A brief review of instruments and techniques. Curr. Pain Headache Rep..

[2] Raja S.N., Carr D.B., Cohen M., Finnerup N.B., Flor H., Gibson S., Keefe F.J., Mogil J.S., Ringkamp M., Sluka K.A. (2020). The revised International Association for the Study of Pain definition of pain: Concepts, challenges, and compromises. Pain.

[3] Zelaya C.E., Dahlhamer J.M., Lucas J.W., Connor E.M. (2020). Chronic pain and high-impact chronic pain among U.S. adults, 2019. NCHS Data Brief.

[4] Nahin R.L., Sayer B., Stussman B.J., Feinberg T.M. (2019). Eighteen-Year Trends in the prevalence of, and health care use for, noncancer pain in the United States: Data from the Medical Expenditure Panel Survey. J. Pain..

[5] Reid M.C., Eccleston C., Pillemer K. (2015). Management of chronic pain in older adults. BMJ.

[6] Tompkins D.A., Hobelmann J.G., Compton P. (2017). Providing chronic pain management in the “Fifth Vital Sign” era: Historical and treatment perspectives on a modern-day medical dilemma. Drug Alcohol Depend..

[7] Domenichiello A.F., Ramsden C.E. (2019). The silent epidemic of chronic pain in older adults. Prog. Neuro-Psychopharmacol. Biol. Psychiatry.

[8] Patel K.V., Guralnik J.M., Dansie E.J., Turk D.C. (2013). Prevalence and impact of pain among older adults in the United States: Findings from the 2011 National Health and Aging Trends Study. Pain.

[9] Gaskin D.J., Richard P. (2012). The economic costs of pain in the United States. J. Pain.

[10] Institute of Medicine (US) Committee on Advancing Pain Research, Care, and Education (2011). Relieving Pain in America: A Blueprint for Transforming Prevention, Care, Education, and Research.

[11] Axon D.R., Bhattacharjee S., Warholak T.L., Slack M.K. (2018). Xm2 scores for estimating total exposure to multimodal strategies identified by pharmacists for managing pain: Validity testing and clinical relevance. Pain Res. Manag..

[12] Axon D.R., Patel M.J., Martin J.R., Slack M.K. (2019). Use of multidomain management strategies by community dwelling adults with chronic pain: Evidence from a systematic review. Scand. J. Pain.

[13] American Chronic Pain Association American Chronic Pain Association (ACPA)-Stanford Resource Guide to Chronic Pain Management 2021 Version. https://med.stanford.edu/content/dam/sm/pain/documents/ACPA-Stanford-Resource-Guide-to-Chronic-Pain-Management-2021-Edition-4-18-21-.pdf.

[14] Tichy E.M., Schumock G.T., Hoffman J.M., Suda K.J., Rim M.H., Tadrous M., Stubbings J., Cuellar S., Clark J.S., Wiest M.D. (2020). National trends in prescription drug expenditures and projections for 2020. Am. J. Health-Syst. Pharm..

[15] Schumock G.T., Stubbings J., Hoffman J.M., Wiest M.D., Suda K.J., Rim M.H., Tadrous M., Tichy E.M., Cuellar S., Clark J.S. (2019). National trends in prescription drug expenditures and projections for 2019. Am. J. Health-Syst. Pharm..

[16] Raggi A., Leonardi M., Mellor-Marsá B., Moneta M.V., Sanchez-Niubo A., Tyrovolas S., Gine-Vazquez I., Haro J.M., Chatterji S., Bobak M. (2020). Predictors of pain in general ageing populations: Results from a multi-country analysis based on ATHLOS harmonized database. J. Headache Pain.

[17] Healthy People General Health Status. https://www.healthypeople.gov/2020/about/foundation-health-measures/General-Health-Status.

[18] Agency for Healthcare Research and Quality Survey Background. https://meps.ahrq.gov/mepsweb/about_meps/survey_back.jsp.

[19] Agency for Healthcare Research and Quality MEPS HC-216 2019 Full Year Consolidated Data Codebook. https://meps.ahrq.gov/data_stats/download_data/pufs/h216/h216cb.pdf.

[20] Agency for Healthcare Research and Quality MEPS HC-216 2019 Full Year Consolidated Data File. https://meps.ahrq.gov/data_stats/download_data/pufs/h216/h216doc.pdf.

[21] Sambamoorthi U., Shea D., Crystal S. (2003). Total and out-of-pocket expenditures for prescription drugs among older persons. Gerontologist.

[22] Axon D.R., Le D. (2021). Predictors of pain severity among community-dwelling older adults with pain in the United States. Medicine.

[23] Axon D.R., Pesqueira T., Jarrell B., Dicochea D. (2021). Correlation of self-reported pain severity and healthcare expenditures in older United States adults. Scand. J. Pain.

[24] Axon D.R., Arku D. (2021). Associations of multiple (≥5) chronic conditions among a nationally representative sample of older United States adults with self-reported pain. Scand. J. Pain.

[25] Marupuru S., Axon D.R. (2021). Association of multimorbidity on healthcare expenditures among older United States adults with pain. J. Aging Health.

[26] World Health Organization (US) (1986). Cancer Pain Relief (US).

[27] Dowell D., Haegerich T., Chou R. (2019). No shortcuts to safer opioid prescribing. N. Engl. J. Med..

[28] Axon D.R., Slack M., Barraza L., Lee J.K., Warholak T. (2021). Nationally representative health care expenditures of community-based older adults with pain in the United States prescribed opioids vs those not prescribed opioids. Pain Med..

[29] Chou R., Deyo R., Friedly J., Skelly A., Hashimoto R., Weimer M., Fu R., Dana T., Kraegel P., Griffin J. (2017). Nonpharmacologic therapies for low back pain: A systematic review for an American College of Physicians Clinical Practice Guideline. Ann. Intern. Med..

[30] Axon D.R., Chien J., Dinh H. (2021). Comparison of health care expenditures among U.S. older adults with pain who reported frequent exercise versus nonfrequent exercise. J. Aging Phys. Act..

[31] Majchrzycki M., Kocur P., Kotwicki T. (2014). Deep tissue massage and nonsteroidal anti-inflammatory drugs for low back pain: A prospective randomized trial. Sci. World J..

[32] Skelly A.C., Chou R., Dettori J.R., Turner J.A., Friedly J.L., Rundell S.D., Fu R., Brodt E.D., Wasson N., Kantner S. (2020). Noninvasive Nonpharmacological Treatment for Chronic Pain: A Systematic Review Update [Internet].

[33] Erstad B.L., Puntillo K., Gilbert H.C., Grap M.J., Li D., Medina J., Mularski R.A., Pasero C., Varkey B., Sessler C.N. (2009). Pain management principles in the critically ill. Chest.

[34] Mobily P.R. (1994). Nonpharmacologic interventions for the management of chronic pain in older women. J. Women Aging.

[35] Titler M.G., Rakel B.A. (2001). Nonpharmacologic treatment of pain. Crit. Care Nurs. Clin. N. Am..

[36] Martinez-Rodriguez A., Cuestas-Calero B.J., Farcia-De Frutos J.M., Marcos-Pardo P.J. (2021). Psychological effects of motivational quativ resistance interval training and nutritional education in older women. Healthcare.

[37] Porter D., Cochran S., Zhu X. (2017). Current usage of Traditional Chinese Medicine for breast cancer- a narrative approach to the experiences of women with breast cancer in Australia—A pilot study. Medicines.

